# Structural testing of concrete walls on-edge with combined axial and out-of-plane loading

**DOI:** 10.1016/j.mex.2023.102182

**Published:** 2023-04-12

**Authors:** Salam Al-Rubaye, Marc Maguire

**Affiliations:** Durham School of Architectural Engineering and Construction, University of Nebraska–Lincoln, 1110 S. 67th St., Omaha, NE 68182, United States

**Keywords:** Tilt-up construction, Second-order loading, Airbag structural testing, Concrete insulated wall panels, Airbag testing of walls in an edge orientation including second-order effects

## Abstract

Full-scale testing of structural components can be time consuming and difficult. The design of full-scale slender concrete walls is highly influenced and controlled by second-order and out-of-plane bending loads. Previous experiments on out-plane bending of slender walls and insulated walls have been performed with bending in the direction of gravity (with or against). Additionally, most of the research considering out-of-plane bending does not include an axial load and suffers from inaccurate results due to not simulating the actual loading and constraining conditions or safety issues. This testing method was developed expressly for the determination of slender wall behavior in insulated concrete panels and verified on solid slender walls, which are well understood. The testing setup presented has the following advantages

• Reduces the risk of cracking panel prior to testing and provides safe and rapid testing.
• Offers ease of implementation in labs with height restrictions, given sufficient floor space.
• Integrates axial and lateral uniform loading.

## Graphical abstract

**Figure:**
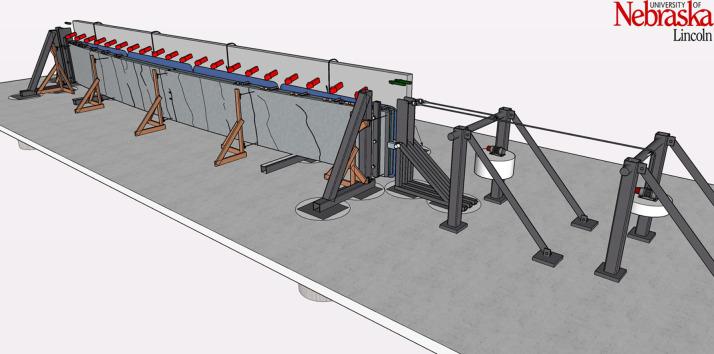

Specifications table

**Table:** 

Subject Area:	Engineering
More specific subject area:	Structural Engineering, Tilt-Up Concrete Engineering
Method name:	Airbag testing of walls in an edge orientation including second-order effects
Name and reference of original method:	None, though several research programs have tested walls in various positions.
Resource availability:	N/A

## Test frame description

**Fig. 1:**
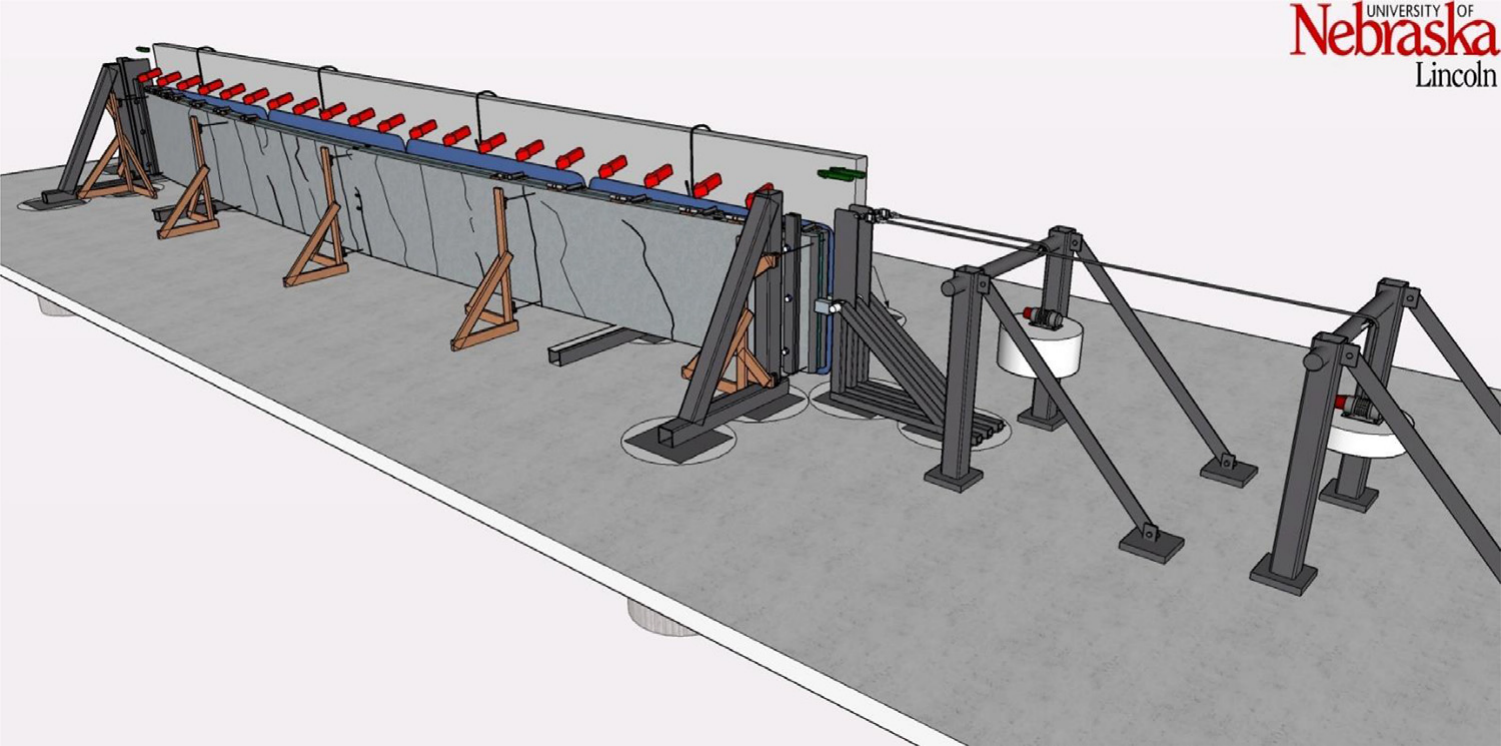
3D drawing of the test setup.

**Fig. 2:**
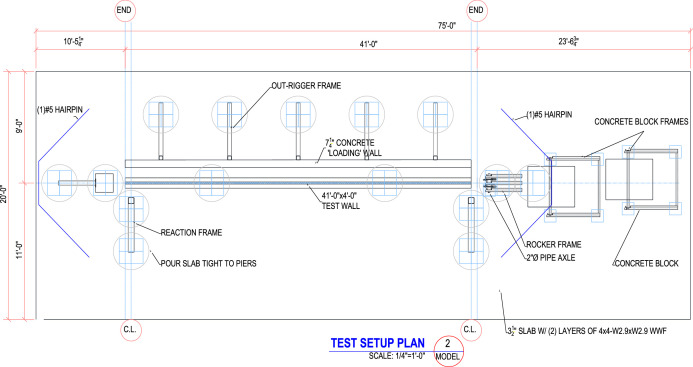
Plan view of test setup for all large-scale panels in this research.

**Fig. 3:**
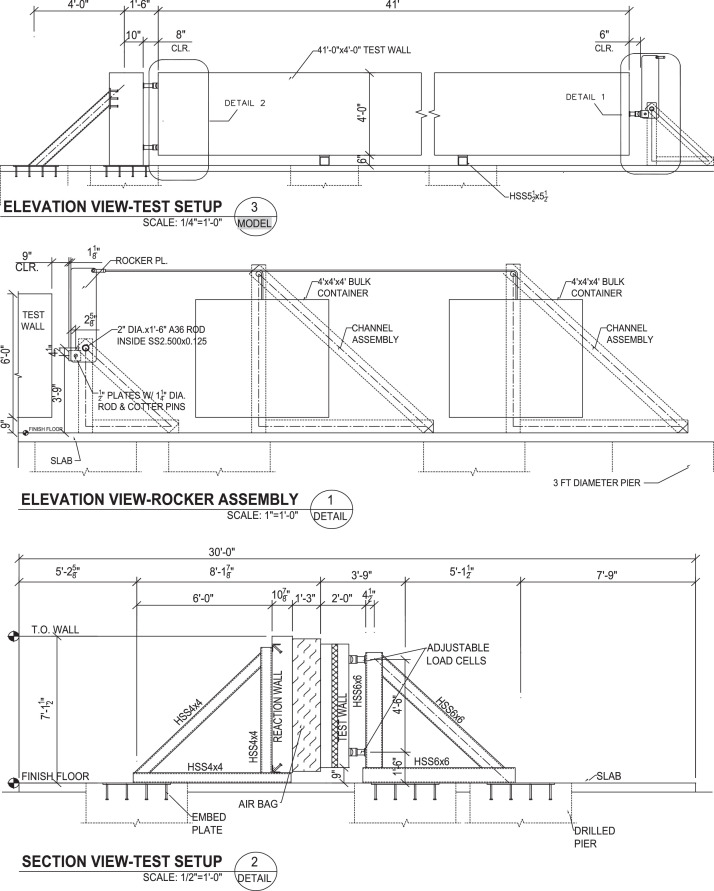
Elevation view of test setup for all large-scale panels in this research.

The large-scale testing portion of this research was comprised of testing slender wall panels in simultaneous out-of-plane flexure and axial compression to study out-of-plane flexure including second order (P-δ) effects. The test apparatus was designed and built solely as part of this project and was a joint effort between the University of Nebraska-Lincoln with Needham DBS, a consulting firm in Kansas City, Kansas, providing all steel material, detailing, fabrication, and engineering to specification [1]. The test setup consisted of three main parts, which are the foundation, the reaction wall with associated abutments, and the gravity load simulator as shown in Fig. 1. The test setup plans are shown in Fig. 2 through Fig. 3.

**Fig. 4:**
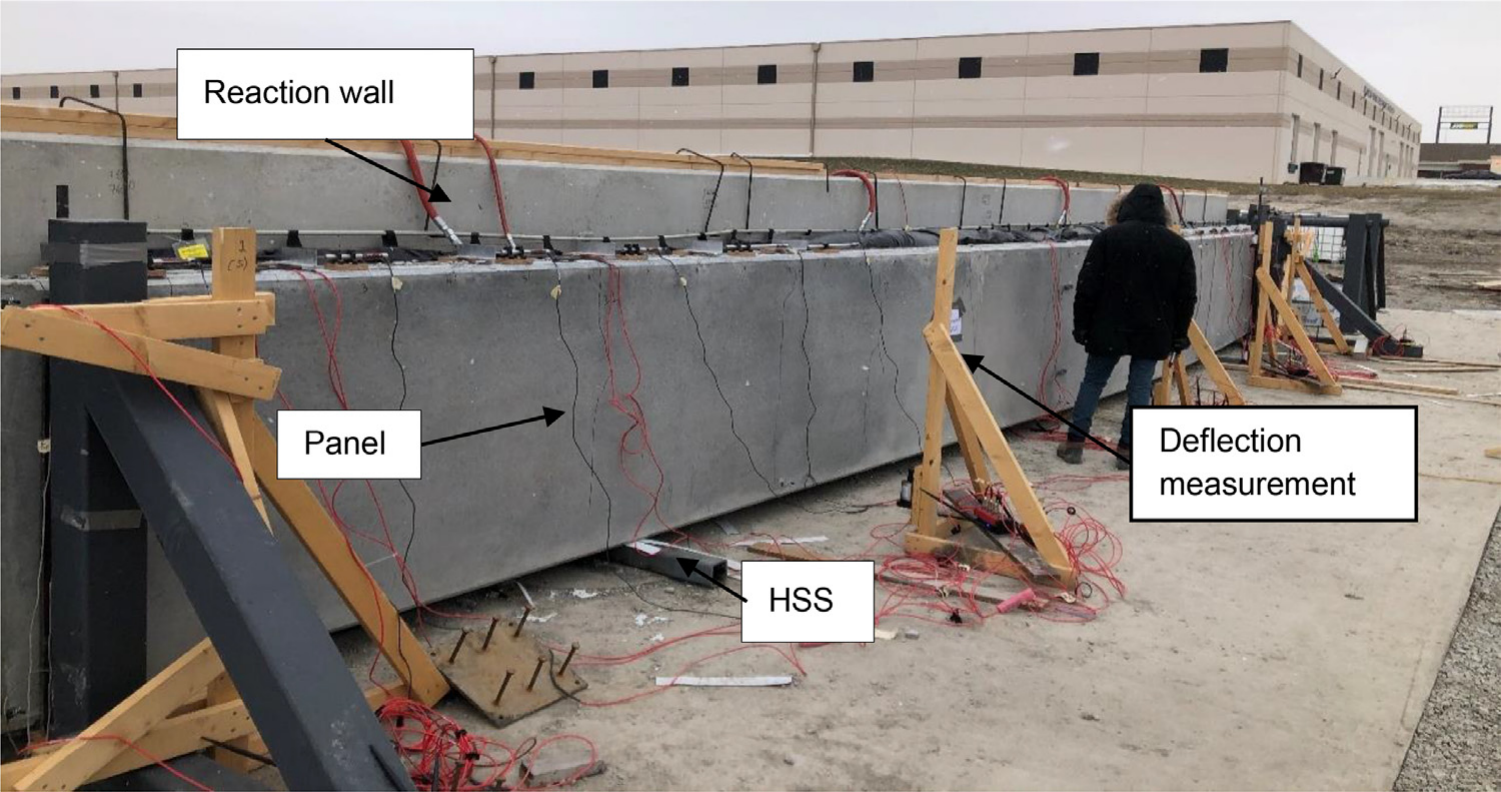
Panel ready for testing.

**Fig. 5:**
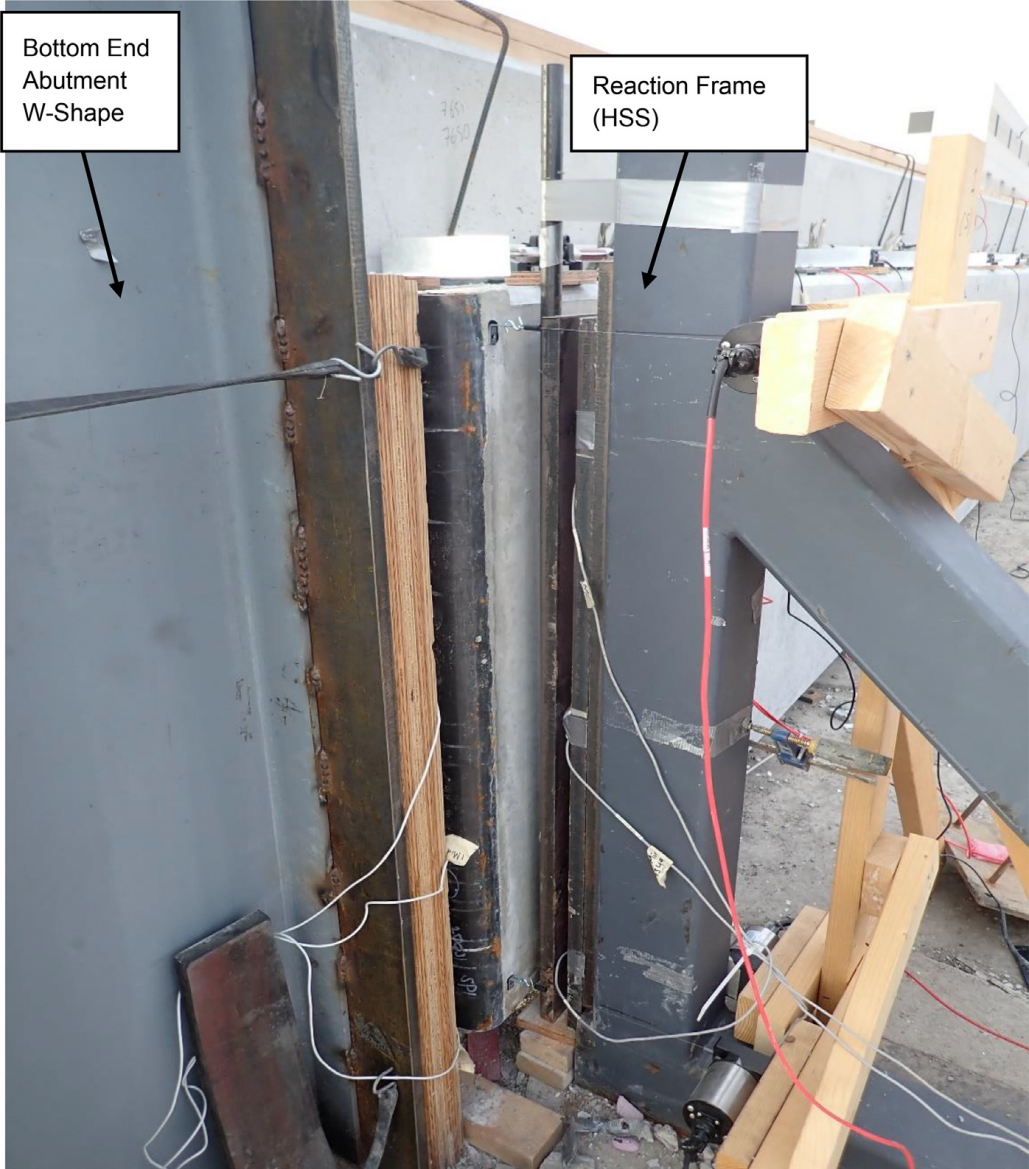
Reaction frame.

**Fig. 6:**
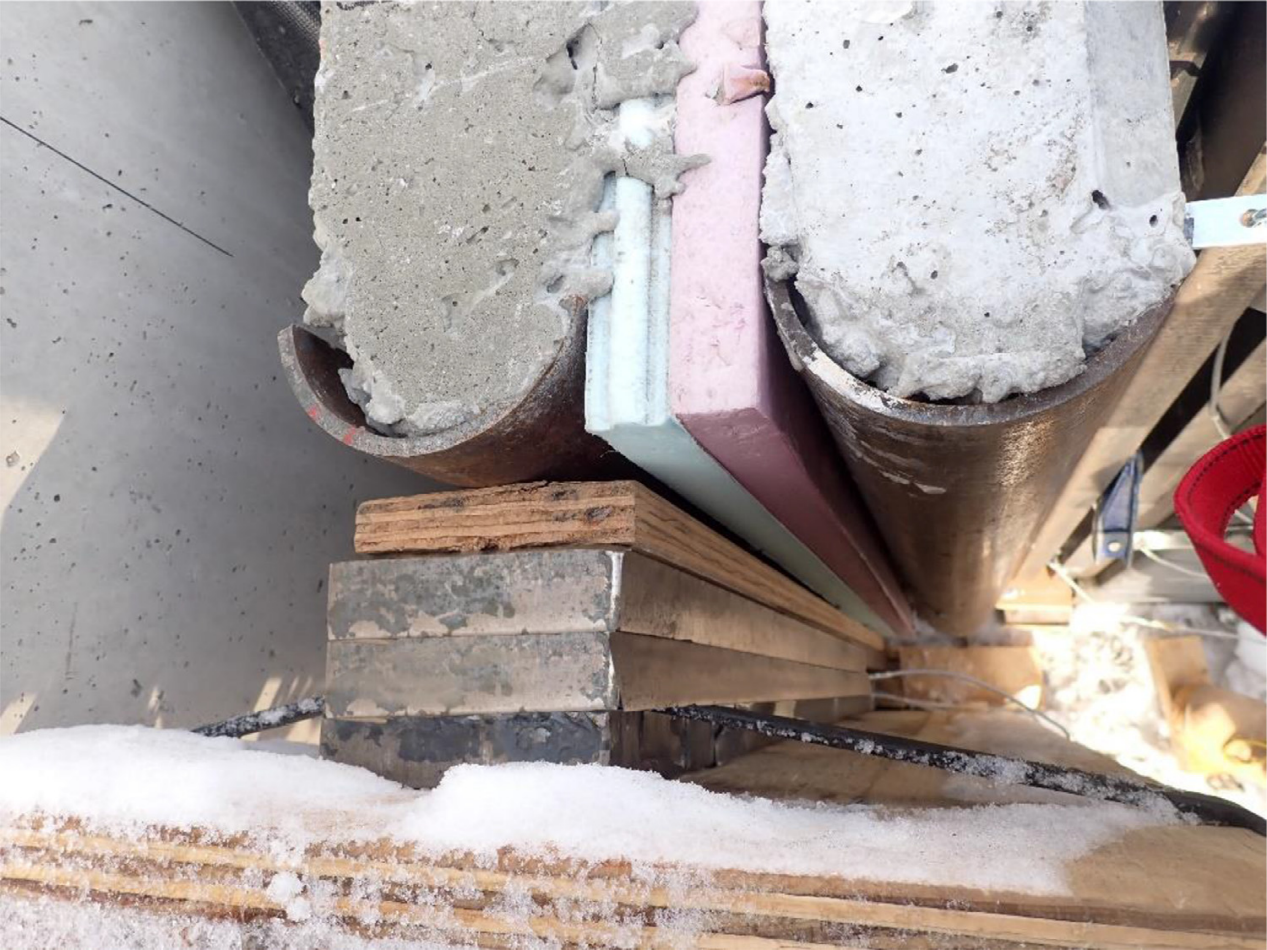
End support.

All metal frame connections to the foundation consisted of short unreinforced concrete drilled shaft piles connected with the 3.5 in. thick reinforced concrete slab with a steel plate embedded for welded connections. A 6 ft by 41 ft by 7.25 in. reaction wall was braced with five total HSS abutments on its back side, whereas its front side was to be used as a reaction for the airbag intended to apply out-of-plane loading (see Fig. 4). Two HSS reaction frames were installed at a separation of 40 ft to provide the top and bottom horizontal reactions on the test panels as shown in Fig. 5. A gap between the reaction wall and the top and bottom simply supported HSS A-frame reaction abutments to allow for insertion of the airbag and test panels. An A-frame reaction abutment was located at the bottom of the panel to take the simulated gravity load reaction (Note for the concrete insulated wall panels (CIP) were only supported on one wythe assuming the facia wythe will be floating in the actual building as shown in Fig. 6). Two total HSS3.5 × 3.5 × 3/8 gravity supports (termed “sleepers” herein) were located 7.75 ft from the ends of the panel to shim up the panel and provide a sliding surface that was further treated with greased double layers of 3 in. wide polytetrafluoroethylene (PTFE) pads to minimize any friction from the dead weight of the test panel on the sleepers. Using lubrication and Increasing the contact pressure by decreasing the PTFE size has a significant effect on the breakaway and reduce the coefficient of friction [2]. Note that the panels were very easy to slide for positioning adjustments using standard pry bars. The sleepers were intended to be located at 0.25 L from each end to minimize stresses at the center of the panel but were mislocated during fabrication. The axial load was applied on the two half-pipes at the top of each panel.

Boundary conditions are important to all tests and designs. ACI 318–19, Section 11.8.2.1 specifies that a wall shall be analyzed as a simply supported member, which is generally a requirement of the use of the Slender Wall Design methodology within this section. For this reason, the tests conducted in the frame used this boundary condition, however, future tests could introduce modified boundary conditions using this same generalized testing procedure. Additionally, ACI 318–19 requires that half of the weight of the wall is applied at the midspan location for the purpose of second-order deformations and stress calculations. Such loads can be applied to panels in this testing setup as needed to demonstrate different behavior. The series of tests conducted and discussed here used a prototype design for panels of realistic design.

For the prototype design that informed the testing loads, the internal axial reaction (at the top of the compression wythe) was 8670 lb to mimic the factored design 3670 lb axial dead plus snow load and the 5000 (1.2 × 4200) lb half-weight of the wythe (simulating the correct dead load at midspan). The exterior reaction (at the top of the tension wythe) was 5000 lb to mimic the other half-weight of the wythe. The loads were applied to the half-pipes using anvils connected to a 10:1 rocker arm assembly anchored to two drilled shafts. The top of the rocker arms were connected using shackles and wire rope attached to a pully over two additional frames to carry concrete blocks that weighed 867 lb and 500 lb for the internal and external reactions, respectively (see Figs. 8 and 10). Tension load cells were installed in-line to obtain the exact load applied. This setup helped apply a constant axial load to the panel without using a hydraulic system which would have trouble maintaining a constant load through the expected large rotations and displacements.

### Loading apparatus

Because orthogonal loads (simulated in-plane gravity and simulated out-of-plane wind) needed to be applied, two systems were used. An airbag system was used to apply simulated wind load in the out-of-plane direction of the panel while a rocker arm system and hanging weights were used for simulated gravity loading in the axial direction of the panel.

### Airbag system

**Fig. 7:**
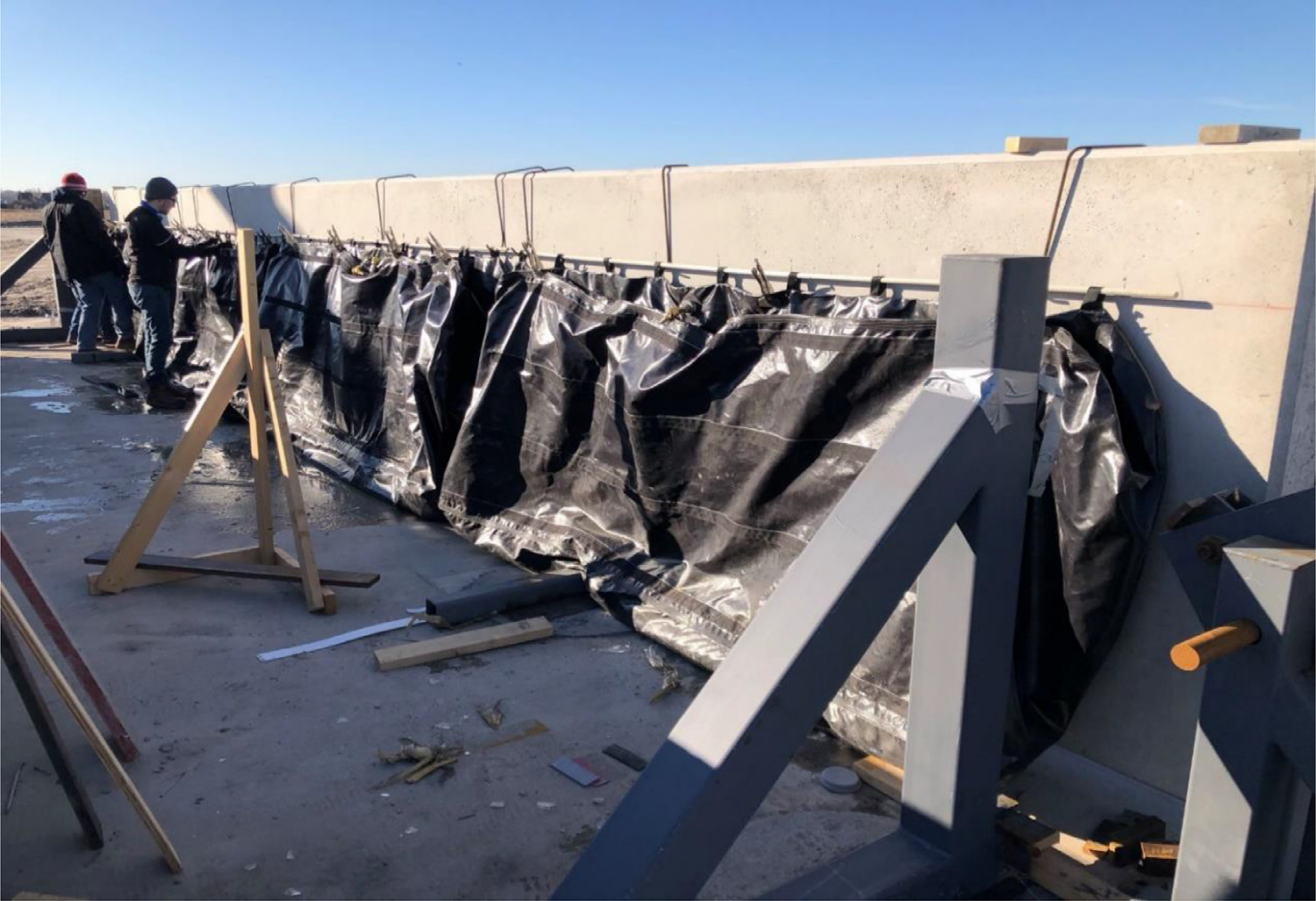
Airbag used during testing.

The simulated out-of-plane wind loading was accomplished with an airbag system. After full vacuum deflation, an assembly of four total 4 ft x 10 ft x 30 in. airbags were installed on the strong wall at the correct height and fixed - as collapsed as possible - using clamps as shown in Fig. 7. Due to the tight clearances, the allotted gap was approximately four in. between the test panel and the strong wall. Each airbag used an air pressure sensor to monitor the internal air pressure of the individual bag. Each airbag was connected with 20 ft hoses to a manifold that was connected to a consumer grade air compressor.

### Rocker arm

**Fig. 8:**
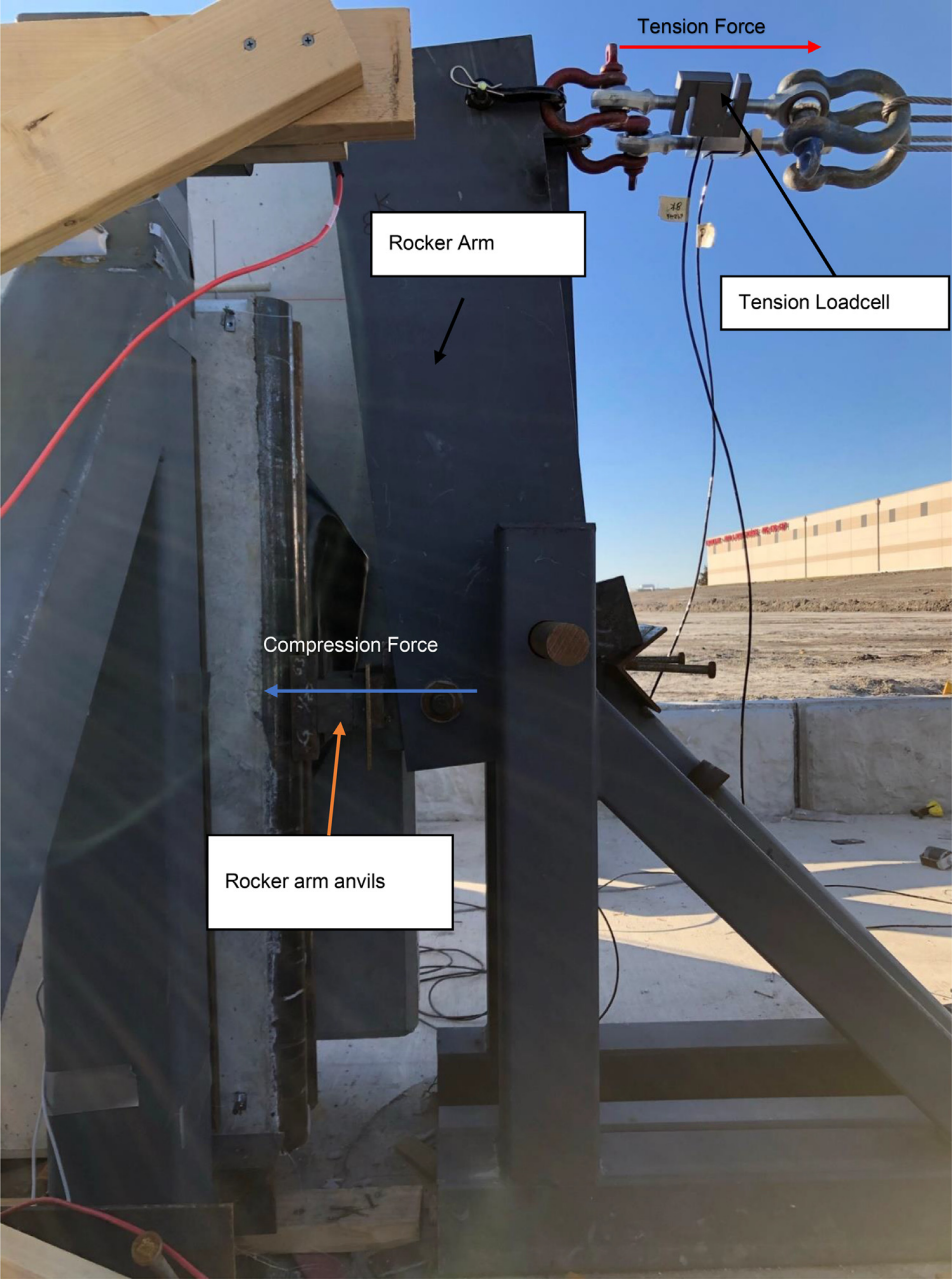
Rocker Arm assembly.

The simulated axial gravity loading was accomplished using a rocker arm and pulley system that hung concrete weights connected to electric winches. The rocker arm assembly was comprised of large steel plates pivoting about a two in. diameter greased steel pin attached to a set of three A-frames connected to two drilled shafts. The load is applied to the half-pipe embedded in the panel through an anvil connected to another pin on the rocker arm enabling the load to maintain consistent contact. A photo of the rocker arm assembly is in Fig. 8.

**Fig. 9:**
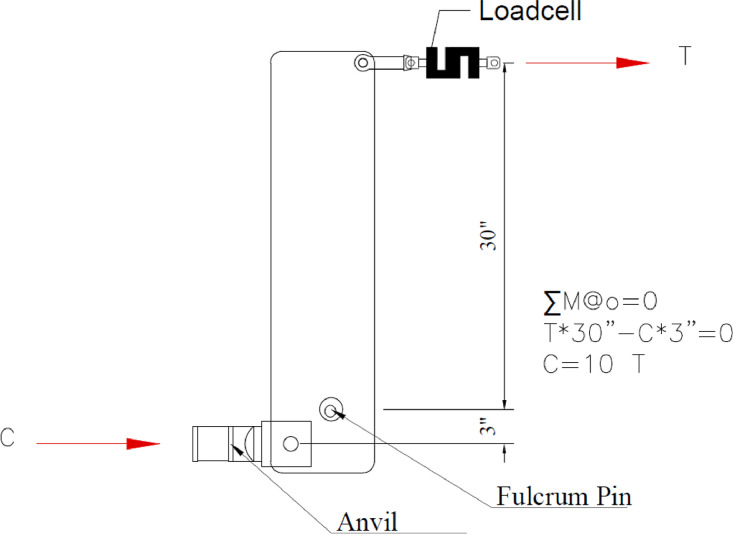
Free Body diagram for the rocker arm.

The rocker arms were designed with a 10 to 1 mechanical advantage, as shown in Fig. 9. S-beam load cells (10 kips maximum, full-bridge, accuracy of +/−0.03% full-scale output) were used to measure the tension applied to the top of the rocker arm as accurately as possible. In early iterations of this loading, the authors attempted to place a load cell between the top of the panel half-pipe and the anvil, but this proved to be too unstable for the duration of the test, with early mis-tests caused by the load cell shooting out. Long slender panels create very large rotations making any such measurement unstable. The fulcrum pin was greased and allowed some movement along its length to accommodate rotational movement at the end of the panel. Small steel channels and steel shims were used between the anvil and the panel half pipes so the rocker arm could be installed in its proper vertical position, as shown in Fig. 9.

**Fig. 10:**
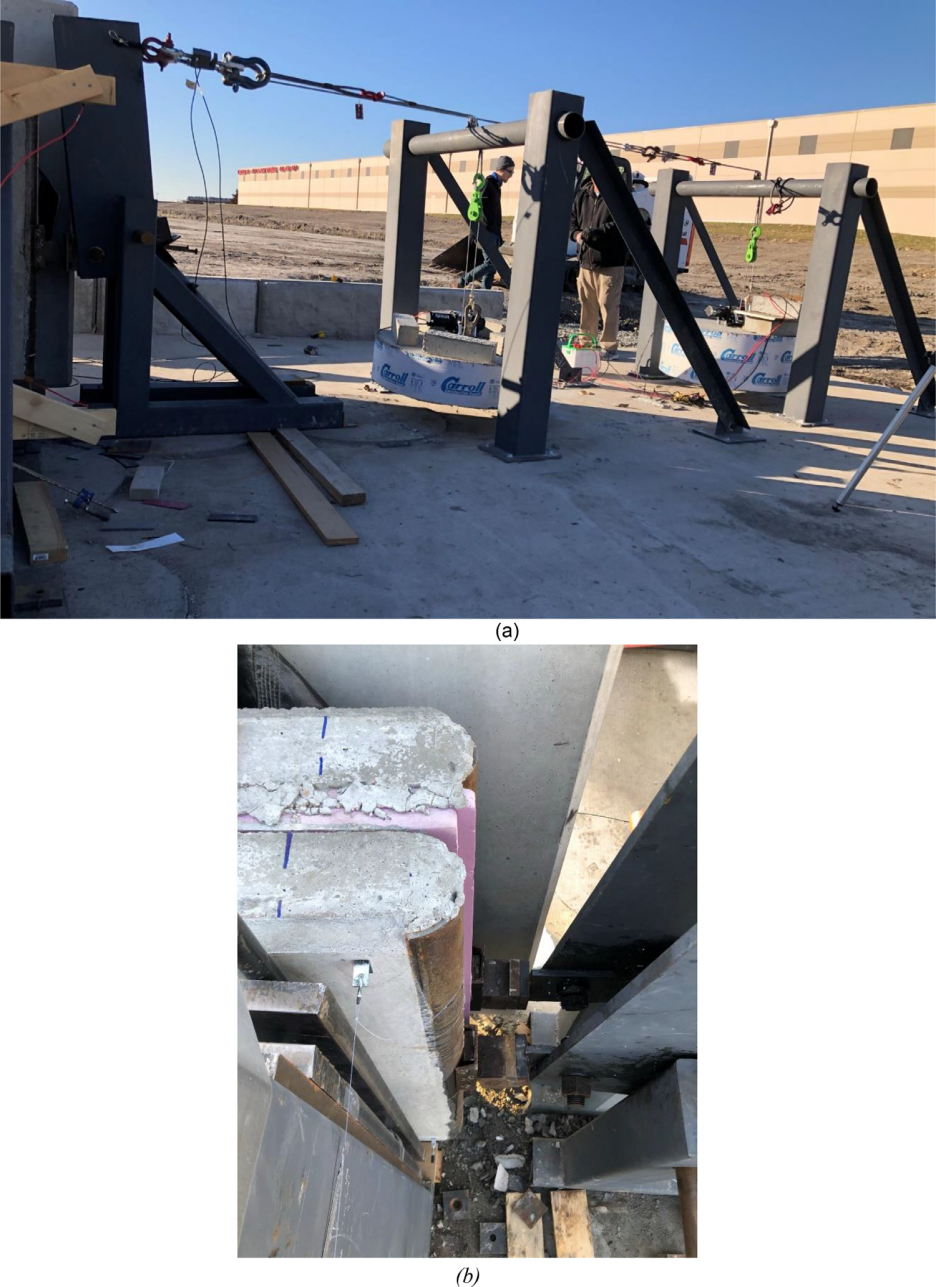
Axial Load (a) concrete blocks, frames, and pulleys used to apply the load and (b) axial load applied on the concrete wythe. (photos from post-failure).

The S-beam load cells were attached to wire rope that was run over a greased pully welded to a pipe and connected to an electric winch and concrete block, as shown in Fig. 10. The concrete blocks were augmented with steel plates to achieve the exact target loading per the load cell readings.

## Monitoring

**Fig. 11:**
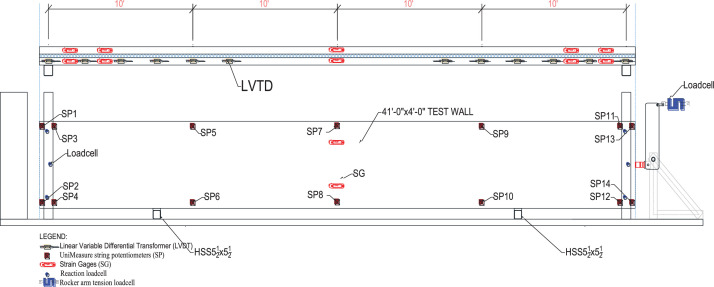
Instrument location.

A full sensing plan for future tests would depend on the goal of the tests to be performed, however, there are specific data points that are required to understand the behavior of the test. For the tests performed by the authors, sensors were installed to monitor various loads or displacements to better understand deformations along the length of the member, as shown in Fig. 11. This section describes the sensors and the purpose of the tests performed as part of this research.

### Instrumentation description

The large-scale panels were instrumented with a total of 54 sensors (14 String Potentiometers, 12 Linear Variable Differential Transducers, 12 Strain gages, 8 Loadcells, 4 VWSG, and 4 airbag pressure sensors). Fig. 11 shows the location of the measurements on the elevation view of the panel. The following sections describe sensor locations and purpose.

### Data acquisition

Two data collection systems were used due to a limitation on the total number of channels and sensor types. The airbag pressure, axial load cells, and VWSG were connected to a Campbell Scientific CR6 datalogger. The remainder of the load cell and displacement sensor data was collected by the Bridge Diagnostics Inc. Wireless Structural Testing System, which is an assembly of four channel nodes that interface with a wireless base station wirelessly networked to a laptop.

### Airbags pressure and load cell

**Fig. 12:**
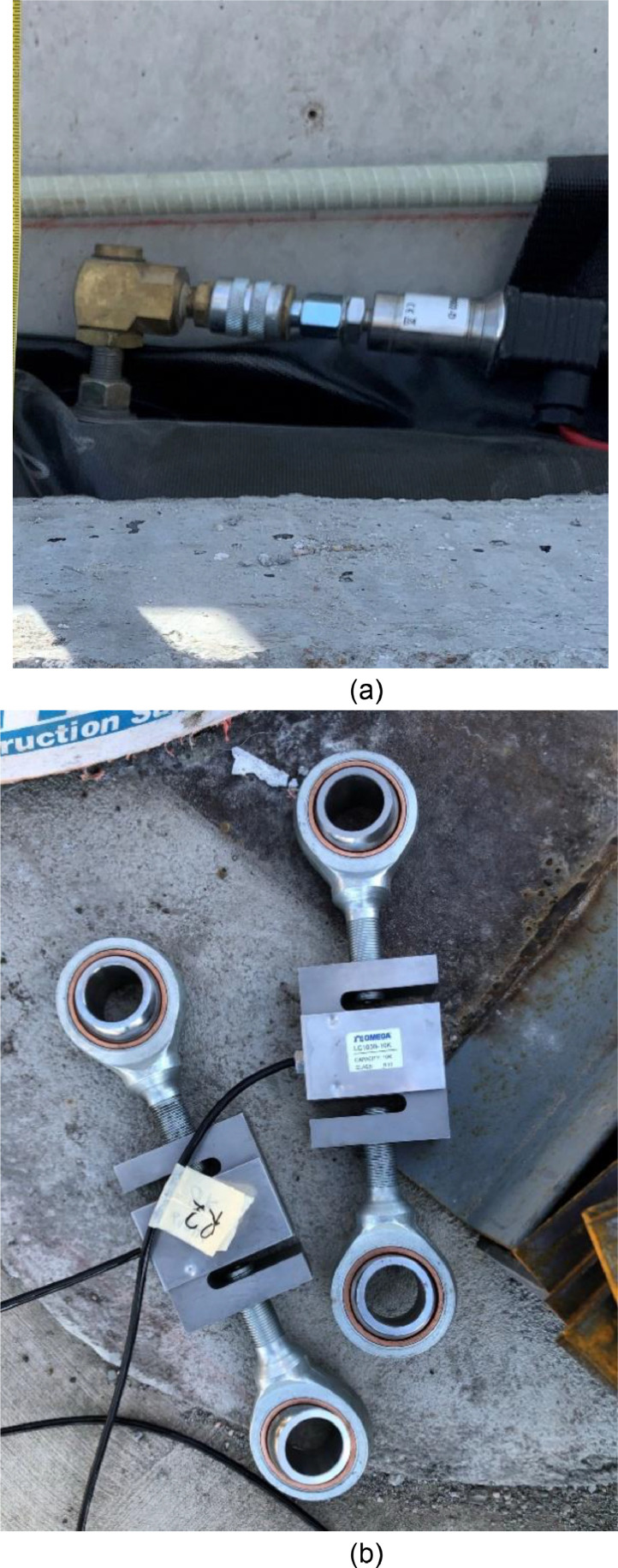
Load and data acquisition devices (a) pressure sensor, and (b) s-beam loadcells.

**Fig. 13:**
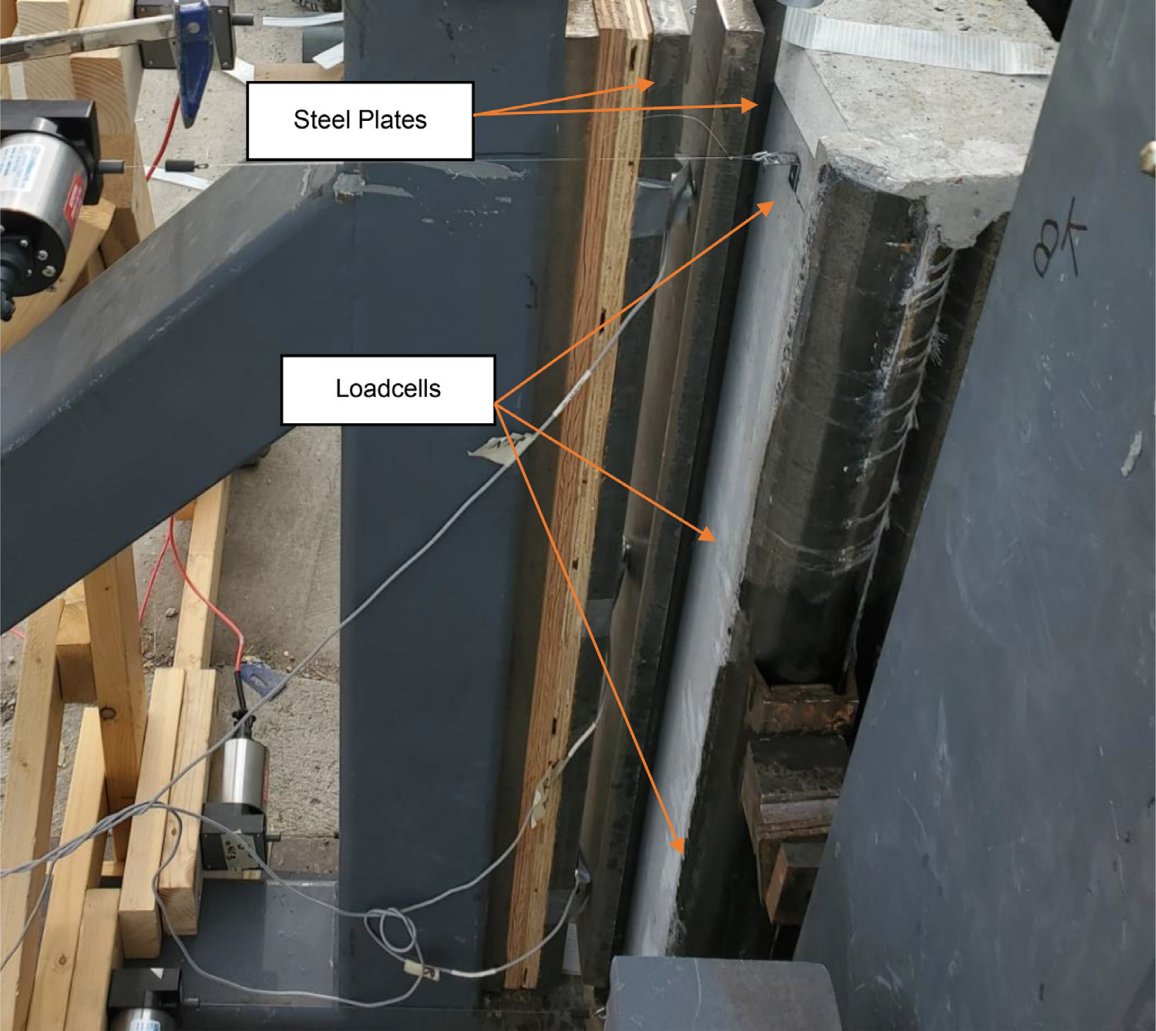
Loadcell location at the reaction frame.

The out-of-plane load was applied using four 4 ft by 10 ft by 30 in. airbags between the reaction panel and the test panel. The airbag pressure was monitored using four total Cynergy3 IPSLU-GP002 (range two psi, accuracy +/−0.25% full-scale output) pressure transducers. Each of the four airbags was monitored independently and was constantly monitored and adjusted to maintain equal pressure using a pneumatic manifold (see Fig. 12). Because the load was of primary importance, load cells were placed between the test panel and the simply supported reactions to the out-of-plane load (see Fig. 13). A trio of Omega LC304 7.5 kip button load cells monitored the support reactions to provide a back-up system for the applied airbag loads. The applied axial loads were measured at the top of the rocker arms using two S-beam Omega LC103B 10,000 lbf load cells in-line with the rigging.

### Deflection measurements

**Fig. 14:**
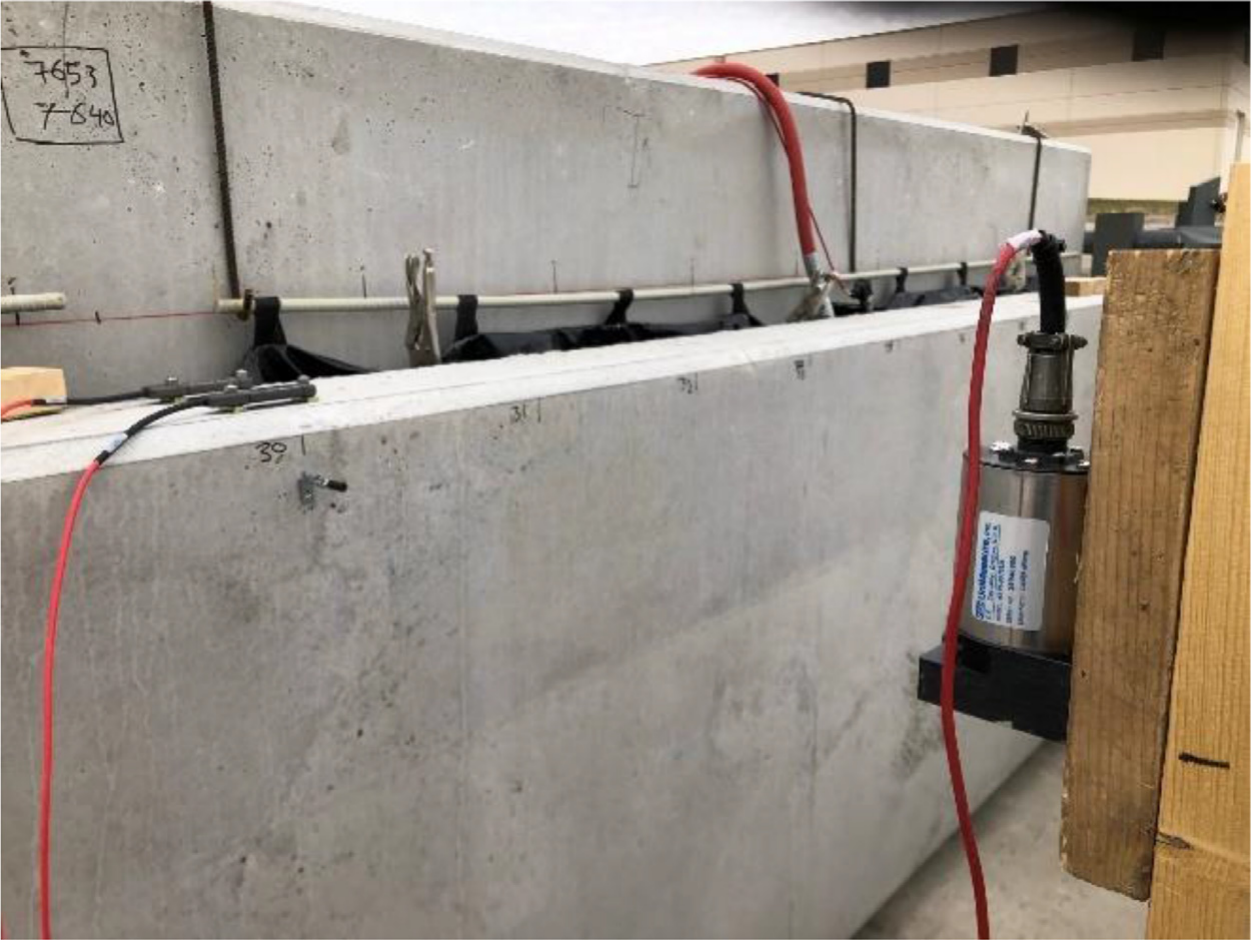
SP on the wood A-frame.

Global displacements were taken at various locations along the plane face of the panel. These displacements were used to determine the deflected shape of the panel. UniMeasure string potentiometers (labeled SP herein; HX series, ranges from 30 in. accuracy ±0.10% full scale and 10 in., accuracy ±0.25% Full Scale) shown in Fig. 14 were used to monitor out-of-plane displacement at the supports and quarter points on the top and bottom edges of the face of the panel. All SPs were mounted to wood A-frames weighed down to the concrete slab to prevent movement or vibration.

### Inter-Wythe relative displacement (Slip) measurements

**Fig. 15:**
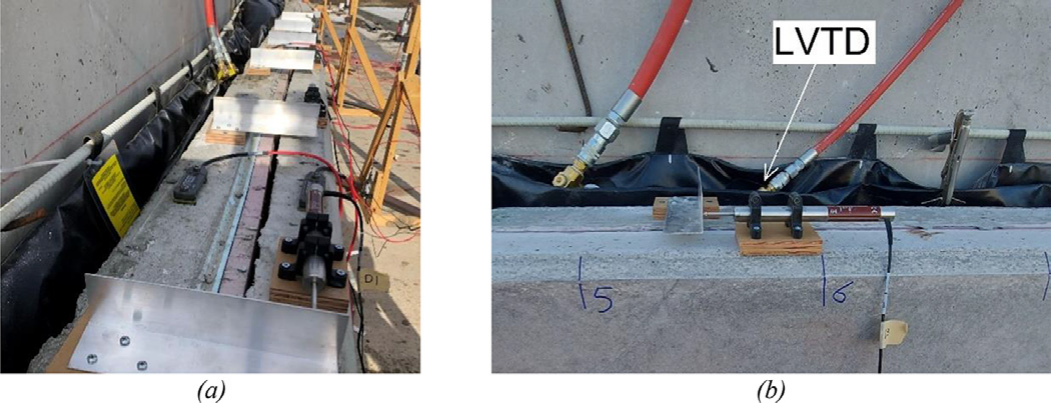
Slip Measurements (a) LVTD along the panel and (b) LVDT.

Linear Variable Differential Transformer (LVDT) displacement transducers measured the relative displacement (slip) between each concrete wythe at twelve discrete locations along the top edge of the panel. An aluminum angle was fastened to one wythe, crossing the insulation layer, while the LVDT was fastened to the other wythe and contacted the angle. Fig. 15 shows the configuration of the slip measuring assembly. Each assembly was located to coincide with a line of wythe connectors. Because the wythe connector locations varied, the location of the assemblies varied for different panel groups.

### Strain sensors

**Fig. 16:**
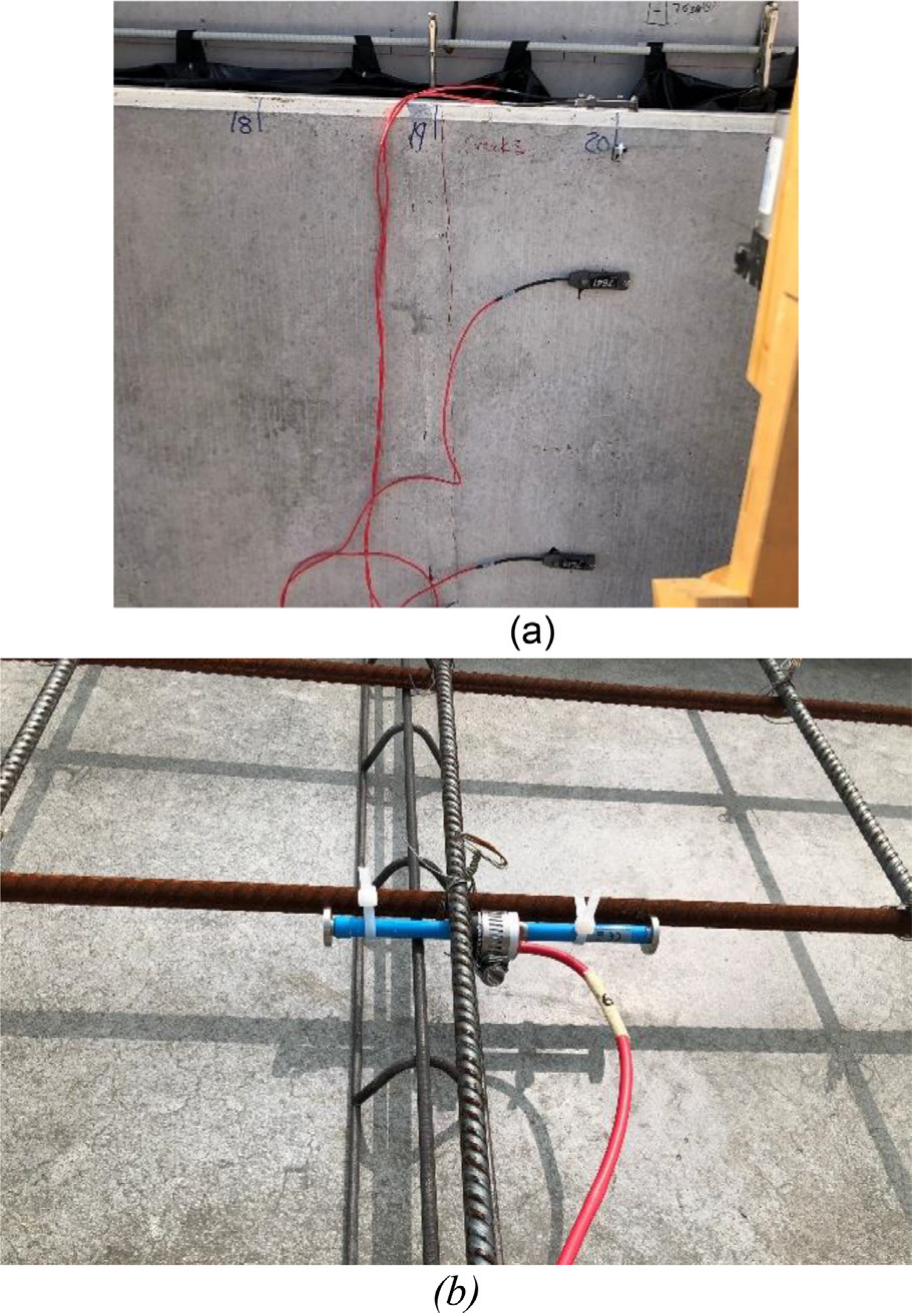
Strain sensors (a) stain transducer on the panel, (b) vibrating wire gages on the rebar prior to casting.

The surface strain was measured at different locations along the panels using the strain transducers presented in Fig. 16. Surface strains were measured using a 3 in. gage length full-bridge BDI ST350 transducer at the locations shown in Fig. 11. Internal strain was measured using GEOKON Model 4200 VWSG as shown in Fig. 16.

### Load sensors

**Fig. 17:**
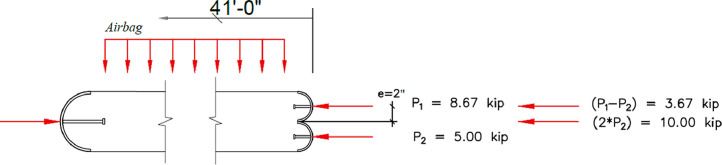
Airbag pressure and Axial load location.

**Fig. 18:**
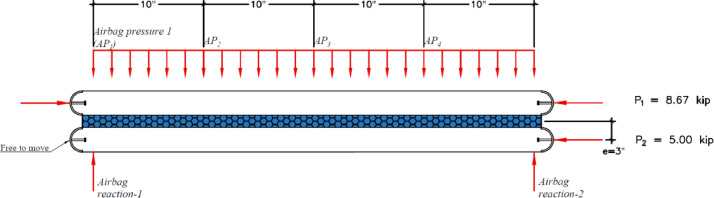
Free body diagram for a concrete insulated wall panel under airbag pressure (not to scale).

The airbag pressure sensors are required to understand the out-of-plane moment imparted. Each bag must be monitored if multiple airbags are used like those herein. Monitoring only at the manifold did not work well in early tests as the individual airbag pressures did not match the dial gage. Each airbag must be controlled manually or electronically to maintain equal airbag pressure. The contact area of the bag was monitored manually using a tape measure throughout the test. For the testing described herein, the contact area remained constant because the airbags were of rectangular prism construction. The total moment resulting from the out-of-plane pressure imparted by the four-bag assembly and the applied axial load can be calculated from the following equation based on a static equilibrium analysis for a solid concrete panel (see Fig. 17) using Eq. (1) and for concrete insulated wall panel (see Fig. 18) using Eq. (2). Note the concrete insulate wall panel (CIP) was supported only on the interior wythe, changing the moment from axial load.

(1) $$M_{total}=M_{w}+\left(P_{1}-P_{2}\right)*e+\left(P_{1}+P_{2}\right)*\Delta$$

(2) $$M_{total}=M_{w}+P_{2}*e+\left(P_{1}+P_{2}\right)*\Delta$$

Where:

*M_total_* = Total Moment (kip*ft)
$M_{w}$ = applied lateral moment from the all-airbag forces on panel, kip*ft
*P_1_* = half of panel weight at mid-height + applied axial load, nominally 8.67 kip, as shown in Fig. 17
*P_2_* = half of panel weight at mid-height, nominally 5 kip, as shown in Fig. 17.
*e* = eccentricity of applied axial load, ft
Δ = deflection at mid-height, ft

The airbag reaction was calculated using Eq. (3) by taking the summation of moments about airbag reaction-2, as shown in Fig. 18. The results from the airbag pressure sensors were verified with the load cell at the reaction frame.

(3) $$R_{Airbag-1}=\frac{1}{40\text{ft}}\left(AP_{1}*A_{\text{AP}1}*\left(\frac{10\text{ft}}{2}+30\text{ft}\right)+AP_{2}*A_{\text{AP}2}*\left(\frac{10\text{ft}}{2}+20\text{ft}\right)+AP_{3}*A_{\text{AP}3}*\left(10\text{ft}+\frac{10\text{ft}}{2}\right)+AP_{4}*A_{\text{AP}4}*\left(\frac{10\text{ft}}{2}\right)\right)\;$$

Where:

$R_{Airbag-1}\;$= Airbag reaction (lbf)
AP = Airbag pressure (psf)
$A_{\text{AP}}$ = contact area of Airbag pressure

**Fig. 19:**
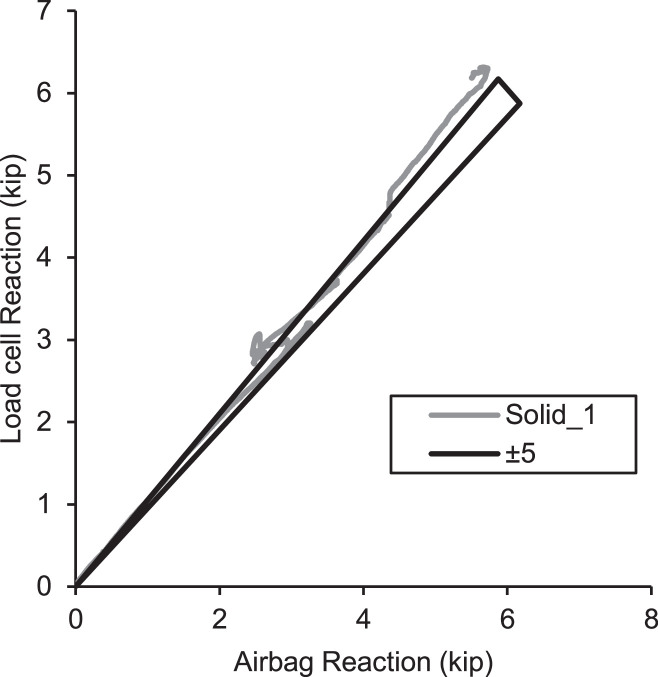
Airbag calculated load vs load cell measurement at the support.

The authors attempted to measure the out-of-plane reaction forces at the simple support locations by using a load cell assembly at each A-frame support reasonably good success. Rollers were installed on top of a tripod of button-type load cells (3kip capacity, accuracy +/- 0.25% full scale) sandwiched between long plates as shown in Fig. 13. The three load cells were an attempt to obtain a stable measurement of the reaction load as the roller moved during loading for verification purposes. However, the movement was too much in some cases as load cells were occasionally overloaded. Additionally, in at least one case, the roller nearly rolled off the assembly due to large displacements and this was later thrown out. Fig. 19 is a plot of the load cell reaction versus the predicted airbag reaction (based on statics from airbag pressures) along with *a* +/−5% bound. When comparing the reactions, it seems that the two methods of load measurement were in reasonable agreement. Given this information, the load cell measurements are not recommended in the future unless modifications are made to the arraignment.

### Displacement sensors

**Fig. 20:**
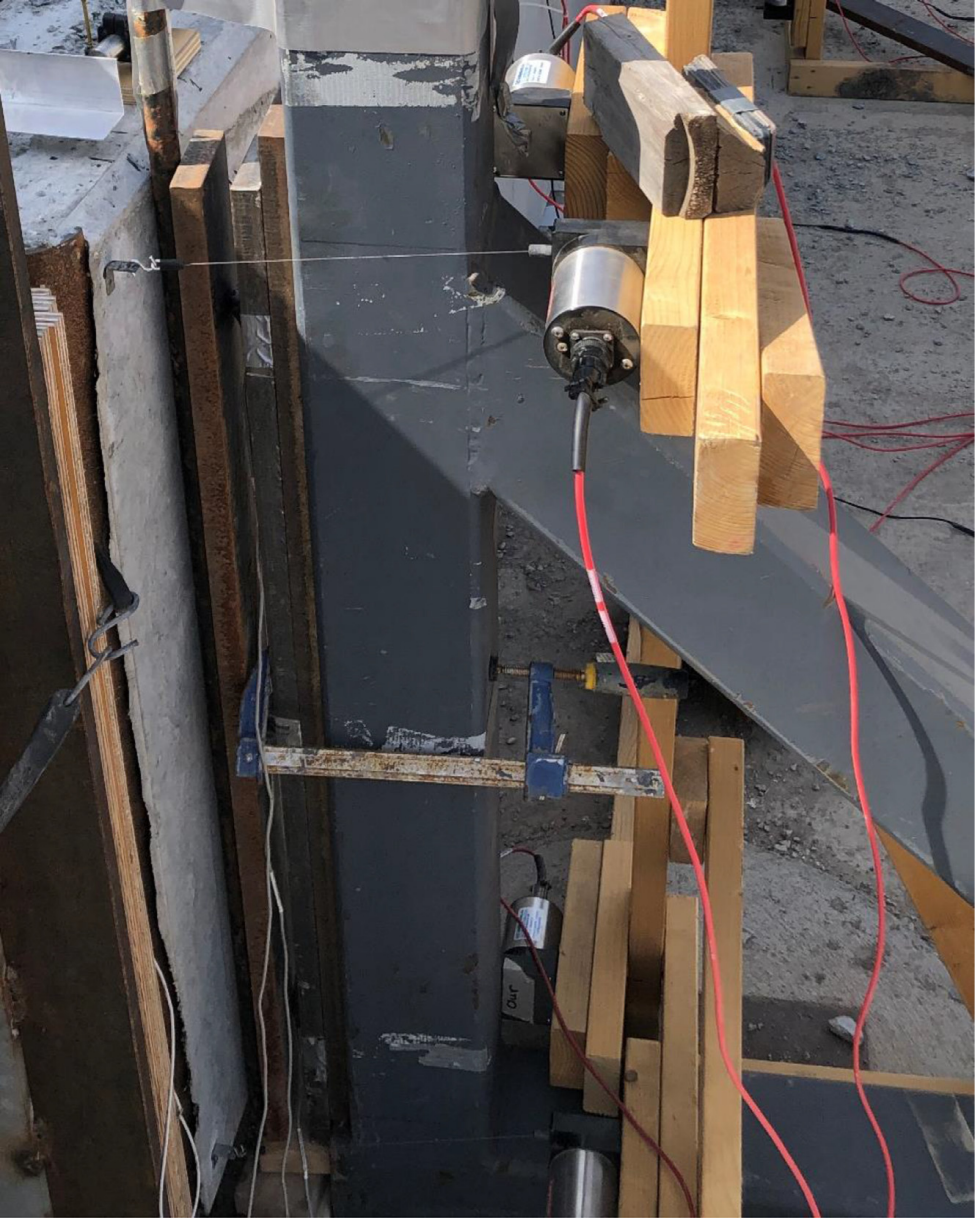
Close view of the SPs at the support.

Out-of-plane displacement is a very important measurement to understand the second-order effects imparted to the wall, particularly the midspan displacement. Measuring midspan displacement at the geometric center of the panel would be insufficient. Any given panel will not sit perfectly vertically due to formwork construction tolerance so it may rotate into the simple supports upon loading. Further, it is impossible to get the panel to sit perfectly, touching the reaction points at the simulated bottom of the panel (opposite the rocker arm) and the out-of-plane load supports. Additionally, while effort was made to construct stiff A-frames for the simple supports, they could deform during loading. For each of these reasons, support displacements must be meticulously measured (see Fig. 20).

**Fig. 21:**
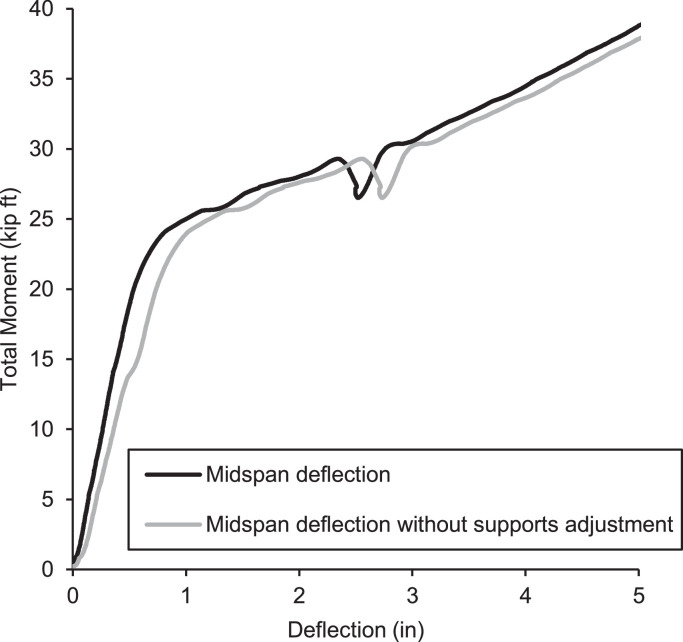
Midspan deflection with and without supports adjustment.

Due to the geometry of the out-of-plane simple supports, wood A-frames were constructed to reference SPs to the ground and measure the displacement at the top long edge and bottom of the long edge at 6 inches from the center of the roller support. This resulted in four total SP displacement measurements suitable for understanding the translation and rotation of the panel as it is loaded and pushed against the supports by the airbag. If the displacement at these supports is not monitored and subtracted from the midspan displacement, the measured midspan displacement will have obvious measurement artifacts in the data and result in larger displacements reported than are realistic as shown in Fig. 21. In this case, even at low loads, displacements increased by as much as 30–40%, which would obfuscate the critical elastic load displacement behavior for slender wall panels.

## Method process

The specific process used to test a wall panel in the configuration described above is as follows:

1. Clean the area.
2. Prepare the airbag by vacuuming all air and clamp such that it is collapsed and stays in place. Clamps must be removed shortly after air begins to flow.
3. Place greased double layer PTFE sheets on sleepers
4. Using a crane, slide the panel into place from the top
5. Install all sensors as appropriate, specifically displacement and pressure sensors.
6. Measure panel as-built dimensions, including wall thicknesses and width along the length.
7. Begin monitoring sensors for pre-test axial loading.
8. Apply dead load using rocker arm assembly to seat the panel into the below reaction. This may significantly shift the panel on the sliding supports. For this reason, the panel will be reloaded in step 8.
9. Ensure all sensors are working as intended, particularly the rocker arm load cells.
10. Begin to collect data for test. The sample rate should be sufficient to capture data for the load rate selected (see item 12).
11. Remove the dead load rocker arm assembly load and *reapply* the dead load rocker arm assembly load ensuring a tight fit by using shimming plates on the anvil. The top of the arms should be near vertical or slightly toward the panel.
12. Start air compressor, letting it reach working pressure.
13. Open the manifold and apply pressure at a rate of 2 psf per minute. This rate is sufficient to ensure a static test. Alternatively, increase pressure to predefined stopping points and hold the pressure while sensors record.
14. Ensure all bags inflate at a similar pressure.
15. Monitor data to ensure bag pressure and deflections are consistent in real-time.
16. Cracks can be marked, measured, and documented throughout loading.
17. Airbag contact area should be recorded using a ruler, tape measure, or other measuring devices at regular intervals.
18. The test can be stopped upon inflation of the airbag up to its maximum displacement or load capacity (30 in. max in the case herein) or the wall has sufficiently failed.
19. Back-up data from data acquisition.
20. Before removing airbag pressure, fully remove rocker arm loads.
21. Open pneumatic manifold bleed valve to remove air pressure and/or insert vacuum to remove air pressure rapidly.
22. Clear away sensors and clean the area.
23. Remove the panel from the testing apparatus.

## Method validation

**Fig. 22:**
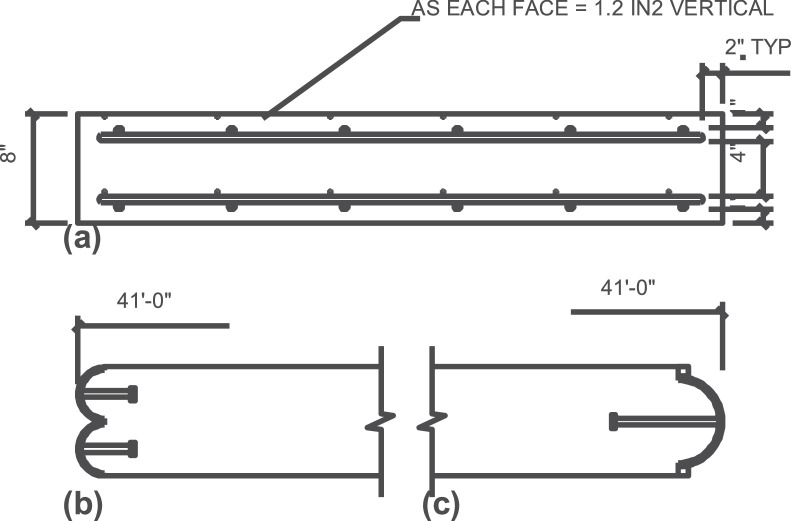
Cross-section of control panel (a) cross section, (b) axail load end assembly, and (c) bottom support end assembly.

**Fig. 23:**
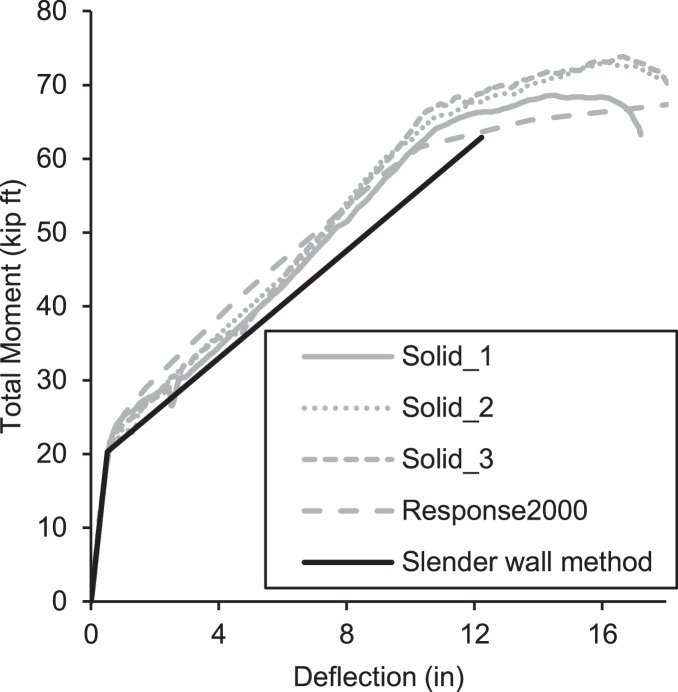
Total Applied Moment versus Deflection for Solid Wall Panels plotted with Response 2000 results and Slender Wall Method.

To assess the accuracy of the testing method, three total 8 in. thick solid section control specimens measuring 41 ft in length and 4 ft wide were fabricated to test load carrying capacity on slender panels as shown Fig. 22. This series of tests (labeled Solid 1, 2, and 3) were intended to allow the researchers to verify the testing setup on a set of specimens where the mechanics were well-known. Solid concrete tilt-up wall panels are very well understood [3,4] and utilize what is known as the Slender Wall Design Methodology [5]. Further, the Response 2000 moment-curvature-deflection program is often used to model the behavior of slender wall panels [6]. The results of solid panel tests are presented as load versus deflection curves using the test setup described herein where the total moment is calculated using Eq. (1), and deflection is determined from the method described in the previous section. Each of these tests is compared to the slender wall method and Response 2000 in Fig. 23.

## Ethics statements

N/A.

## Declaration of Competing Interest

The authors declare that they have no known competing financial interests or personal relationships that could have appeared to influence the work reported in this paper.

## Figures and Tables

**Fig. 24:**
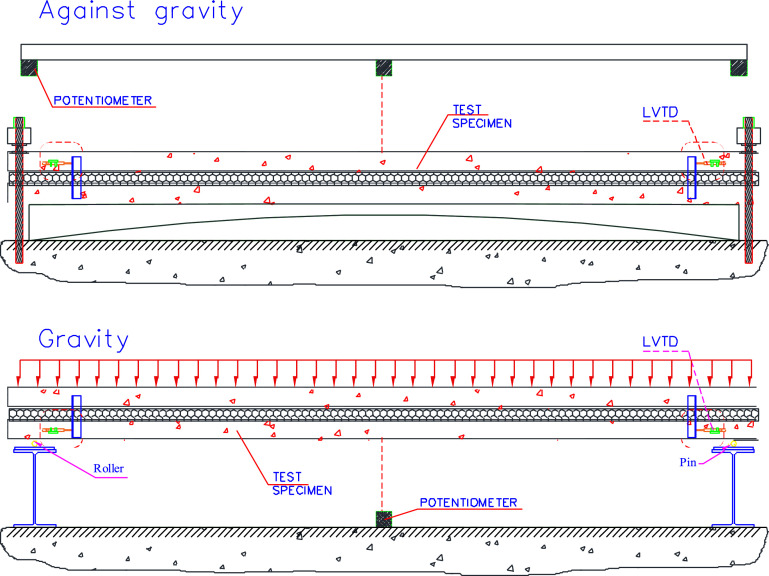
Loading the panel with and against gravity (gravity acts downward in both cases).

## Data Availability

Data will be made available on request.

## Additional information

### Context

Concrete walls of realistic height and construction type in US construction practice are difficult for most structural laboratories. The typical concrete wall for warehouse-style structures continues to increase in height for both site-cast and plant-cast precast. The typical wall is in excess of those tested previously [7] and is anecdotally at 40 ft and taller. Composite insulated concrete walls in particular have a length/height dependence on their behavior due to the types of connectors used to span between the concrete layers [8,9]. Testing walls in an upright condition requires strong frames and auxiliary structures that are very large and stiff that must also be oriented in a vertical direction. This condition also introduces safety issues (worker vertigo, fall protection, etc.) that must be addressed with man-lift and proper safety. If performing tests to failure, vertically oriented panels could be difficult and dangerous to deconstruct.

Many research programs testing precast walls have tested panels flat and pushed against or with gravity to provide out-of-plane loading [9], [10], [11]. Testing flat with applied loading in the direction of gravity risks cracking the panel prior to obtaining meaningful measurements and can require the panel to be overdesigned such that it is not realistic [8] and difficult to account for self-weight. Most wall panels of reasonable heights and practical designs will crack under their own self-weight when loaded in a simple span condition [10,11]. Testing with applied loading against gravity has mixed results for low loading and also seems very difficult to account properly for the self-weight [12] (see Fig. 23, Fig. 24).

The research method described below was intended for testing solid tilt-up wall panels and concrete insulated wall panels under combined axial and lateral loads but could be modified for similar wall types and geometries. Panels were tested with the supports on their long edge with simulated wind loading applied out-of-plane to the flat face using a monotonically increasing airbag load and constant axial load applied using concrete blocks and rocker arms. All panels were reinforced with mild steel and the wythes were connected using fiber-reinforced polymer (FRP) or metal ties. The concrete employed in the specimens' fabrication was also sampled and measured by an ACI-certified technician for compression and bending strengths according to the relevant ASTM standards. Additionally, the reinforcement was tested in tension to get its stress versus strain behavior. Such testing is standard for large scale testing, but it is itemized for completeness.
